# Immunization against full-length protein and peptides from the *Lutzomyia longipalpis* sand fly salivary component maxadilan protects against *Leishmania major* infection in a murine model

**DOI:** 10.1016/j.vaccine.2017.10.039

**Published:** 2017-12-04

**Authors:** William H. Wheat, Erik N. Arthun, John S. Spencer, Daniel P. Regan, Richard G. Titus, Steven W. Dow

**Affiliations:** a Department of Microbiology, Immunology, Pathology, College of Veterinary Medicine and Biomedical Sciences, Colorado State University, Fort Collins, CO 80523, United States; b Department of Biology, College of Natural Sciences, Colorado State University, Fort Collins, CO 80523, United States; c Department of Clinical Sciences, College of Veterinary Medicine and Biomedical Sciences, Colorado State University, Fort Collins, CO 80523, United States

**Keywords:** Leishmania, Vaccine, Sand flies, Saliva, Maxadilan, Adjuvants, Cationic Lipid DNA complexes, CLDC

## Abstract

Leishmaniasis is an arthropod vectored disease causing considerable human morbidity and mortality. Vaccination remains the most realistic and practical means to interrupt the growing number and diversity of sand fly vectors and reservoirs of *Leishmania*. Since transmission of *Leishmania* is achieved exclusively by sand fly vectors via immune-modulating salivary substances, conventional vaccination requiring an unmodified host immune response for success are potentially destined to fail unless immunomodulatory factors are somehow neutralized. Using cationic liposome DNA complexes (CLDC) as an adjuvant system along with *Lu. longipalpis* sand fly salivary component maxadilan (MAX) as antigen (Ag), we show that mice are protected from the MAX-induced exacerbation of infection with *Leishmania major* (*Lm*). The CLDC adjuvant and alum were comparable in terms of lesion induration and decreased parasite burden, however the alum adjuvant imposed more inflammation at the injection site. BALB/c, C3H and C57BL/6 mice vaccinated with MAX-CLDC containing either the full-length MAX or peptides spanning the N- and C-terminal regions of MAX are protected against footpad challenges with *Lm* co-injected with MAX. When compared to unvaccinated controls, all strains of mice immunized with CLDC containing either peptides encompassing the first 20 N-terminal AA or those spanning the last 15 AA of the C-terminal domain of MAX demonstrated decreased parasite burden after 9 or 18 weeks post challenge with *Lm* + MAX. MAX-CLDC immunized mice showed increased IFNγ-secreting and decreased IL-4-secreting CD4^+^ cells in footpad-draining lymph nodes. Antisera from C-terminal peptide (P11) MAX-CLDC-vaccinated animals was capable of recognizing FL-MAX and its C-terminal domain and also blocked MAX-mediated reprogramming of bone marrow-derived dendritic cells (BM-DC) in vitro. This peptide vaccine targeting sand fly MAX, improves host immunity against MAX-mediated immunomodulation.

## Highlights

• Vaccination against maxadilan (MAX) protect mice from aggravated *Leishmania* infection.
• Vaccination against MAX increases the number of IFN-γ-producing cells in draining lymph nodes.
• The peptide MAX-CLDC vaccine improves host immunity against MAX-mediated immune modulation.

## 1 Introduction

Vector-borne disease continues to pose significant global morbidity and mortality accounting for more than 17% of all infectious diseases causing more than 1 million deaths annually [1]. Many of these diseases continue to re-emerge in former endemic areas and/or emerge in new parts of the world where conventional means of control are often inadequate due to the emergence of pesticide-resistant vectors, drug-resistant pathogens, and collapse of vector control programs. At present, safe and effective vaccines and therapeutics for prevention and treatment for many of these conditions are inadequate or all together lacking. Such is the case for leishmaniasis. The leishmaniases are a group of zoonotic vector-borne diseases caused by infection with obligate intracellular protozoa of the genus *Leishmania,* transmitted by infected female sand fly vectors of the genera *Phlebotomus* and *Lutzomyia.*

The saliva of blood-feeding insects has abundance of pharmacologically active components that serve individually or collectively to usurp the host hemostatic system likely evolved to optimize the acquisition of a blood meal and fecundity [2], [3]. In addition to their critical function of aiding sand fly feeding via various hemostatic effects, salivary components such as the vasodilator maxadilan (MAX) from *Lutzomyia longipalpis* have profound immunosuppressive and anti-inflammatory effects, properties that have been attributed to permitting parasite entry into hosts by localized commandeering of host immunity [4], [5]. Since transmission/infection with *Leishmania* parasites is always in the context of saliva, conventional anti-leishmania vaccines that deploy merely parasite antigens (Ag) may have the potential to fail because they depend on non-subjugated host immune responses.

A crucial argument for the development of a saliva-based vaccine for leishmaniasis is the fact that prior exposure to uninfected sand fly bites and/or vaccination with various immunogenic components of sand fly saliva has been shown to confer protection from Leishmaniasis and that the majority of those who recover from the disease have lasting immunity against salivary proteins [6], [7], [8], [9]. In the past, we have shown that injection of *Leishmania major* (*Lm*) admixed with the *Lutzomyia longipalpis* sand fly salivary peptide maxadilan (MAX), can substitute for whole saliva exacerbating infections in terms of induration of lesion and parasite burden while vaccination against MAX is protective against infection with *Lm* in the context of vector saliva. Furthermore, in the case of disease transmission by *Lu. longipalpis*, MAX may be the major exacerbative element since vaccinating against this molecule neutralized the effects of whole saliva [10].

The work described herein demonstrates that the synthetic full length (FL) MAX molecule as well as C and N terminal peptides derived thereof can be utilized successfully as antigens in a cationic lipid DNA complex (CLDC) adjuvant vaccine system protecting three strains of mice (representing murine susceptibility models ranging from extremely susceptible to completely healing strains) from exacerbation and potentiation of *Lm* infection. Furthermore, we have located a single peptide (P11) comprising the C-terminal 15 AA in MAX that, in the context of the CLDC adjuvant, effectively neutralizes the disease-enhancing effect of MAX.

## 2 Materials and Methods

### 2.1 Reagents

Antibodies used for flow cytometry were: FITC- and/or PE-conjugated anti-mouse CD11c, MHCII, CD86, CD3 and CD4, APC-conjugated anti-mouse CD8a (Ly-2), FITC-conjugated anti-mouse IFN-γ and FITC-conjugated anti-mouse IL-4 (eBioscience, San Diego, CA). Fc receptor block was purchased from Miltenyi Biotec, San Diego, CA. The pituitary adenylate cyclase-activating peptide (PACAP) receptor antagonist PACAP-(6-38) was obtained from Bachem (Heidelberg, Germany).

### 2.2 Mice

5–6 week old (25 g) female BALB/c, C3H-HeN and C57BL/6 mice were obtained from National Cancer Institute (Frederick, MD). Mice were maintained at the Laboratory Animal Resources facility at CSU, Fort Collins, CO. Animal maintenance and care complied with National Institutes of Health Guidelines (under pathogen-free conditions) for the humane use of laboratory animals and institutional policies as described in the American Association of Laboratory Animal Care and Institutional Guidelines. Animal protocols and procedures were approved by the Colorado State University Institutional Animal Care and Use Committee (protocol # 12-3413A).

### 2.3 Lm challenges

Metacyclic promastigotes, from stationary phase promastigotes of *Lm* (LV39 (MRHO/Sv/59/P)) were purified using peanut agglutinin and used for all challenges as described previously [11], [12].

### 2.4 Synthetic maxadilan and MAX peptides

**Supplementary Fig. 1:**
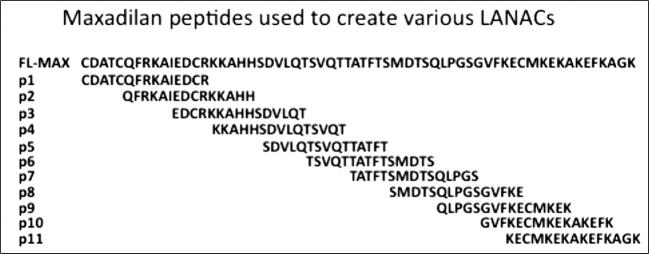
Synthetic MAX peptides used in CLDC formulations. 15-amino acid (AA) peptides were chemically synthesized and used together with the CLDC adjuvant. Each peptide, shown in single-letter code, overlapped their succeeding peptide by 10 AAs and spanned the entire length of the MAX molecule. P11 overlaps P10 by 12 AA.

Synthetic full-length maxadilan and 15 AA over-lapping peptides thereof were prepared by Twenty-first Century Biochemicals, Inc. (Marlboro, MA). The 63 amino acid sequence used was based on the sequence of mature, secreted MAX [13].

(CDATCQFRKAIEDCRKKAHHSDVLQTSVQTTATFTSMDTSQLPGSGVFKECMKEKAKEFKAGK) (Supplemental Fig. S1).

### 2.5 Monitoring lesion development and parasite burden in footpads

**Supplementary Fig. 2:**
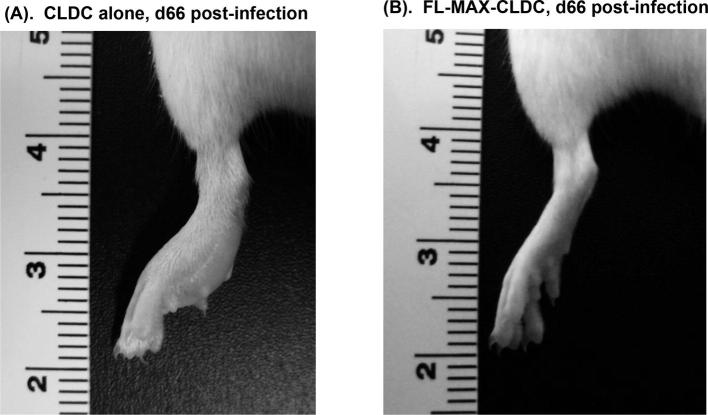
Immunization with FL-MAX-CLDC prevents increase in footpad swelling size following challenge with *Lm* + MAX. BALB/c mice (*n =* 5 animals) immunized with either CLDC or FL-MAX-CLDC were challenged subcutaneously in the left hind footpad with 1 x 10^3^*Lm* metacyclic promastigotes + 10 ng synthetic MAX, and measurements of the effects of immunization on footpad lesion size were determined. Footpad lesion size was determined by measurement of footpad swelling using a digital caliper as described in Materials and Methods. Comparison of representative footpad lesions of mice immunized with (A) CLDC alone vs. mice immunized with (B) the MAX-CLDC reveals significant reductions in cutaneous lesion sizes of MAX-CLDC immunized mice (2 to 3-fold smaller than those of mice receiving CLDC adjuvant only). Images are representative of results obtained from 5 independent experiments. (A): CLDC-immunized BALB/c mouse footpad, day 66 post-challenge. (B): MAX-CLDC-immunized BALB/c mouse footpad, day 66 post-challenge.

Lesion development was followed by measuring increased thickness of infected footpads with a Vernier^®^ caliper and comparisons made between the footpads of the contralateral and of unchallenged controls (Supplemental Fig. S2). Parasite numbers in infected footpads were determined using a technically reliable published limiting dilution assay for *Lm* infection in mice [14].

### 2.6 Preparation of CLDC and alhydrogel® adjuvants and combination with MAX Ags

Cationic liposomes were prepared as previously described by combining equimolar amounts of DOTIM [octadecanoyloxy(ethyl-2-heptadecenyl-3-hydroxyethyl) imidazolinium chloride] and cholesterol [15]). Cationic liposome-DNA complexes (CLDC) were prepared fresh immediately prior to injection by gently mixing cationic liposomes with 100 µg/ml of plasmid DNA (non-coding pDNA, vector 75.6) in 1.0 ml sterile 1mM Tris-buffered 5% dextrose in water at room temperature [16] along with either 50 µg of FL-MAX or 5–50 µg each of 11 peptides spanning the entire 63 AA length of MAX. To prepare the aluminum hydroxide (alum) vaccine, 50 µg of FL-MAX was admixed with 2% (w/v) Alhydrogel^®^ (InvivoGen, San Diego, CA) at 2 mg FL-MAX per mg of alum in phosphate buffered saline. The mixture was allowed to rock for 60 min on rocking platform at RT and administered to mice within 3 h.

### 2.7 Immunizing against MAX and Lm challenges

MAX-CLDC vaccine candidates (FL-MAX or MAX peptides admixed with CLDC) were injected s.c. (two-50 µl injections) into the proximal base-of-tail regions. Two weeks later, the mice were boosted in the same manner. For adjuvant comparison experiments, mice were injected with 5–50 μg of synthetic MAX admixed with Alhydrogel^®^ aluminum hydroxide gel adjuvant (Brenntag Biosector, Frederikssund, Denmark). Other groups of control mice (*n* = 5–8) were immunized with CLDC complexed with the irrelevant control antigen, hen egg lysozyme (HEL), (Sigma Aldrich, St. Louis, MO), or were sham-injected with adjuvant or antigen alone. Fourteen days later, the mice were boosted in an identical fashion. Two weeks following the boost, mice were challenged with a low *Lm* dose (10^2^ to 10^3^) with or without 10 ng MAX.

### 2.8 Anti-MAX ELISA

Blood was collected from tail bleeds at 18 weeks following *Lm* challenge, and the anti-MAX serum titer was determined by ELISA. Briefly, ELISA plates were coated with synthetic FL MAX (10 μg/ml) or MAX peptides (2 μg/ml) using standard techniques [17], [18]) and developed with horseradish peroxidase (HRP)-conjugated goat anti-mouse Ig that detects Ig classes/isotypes (IgG, IgM, IgA polyvalent) (Sigma Aldrich, St. Louis, MO), and developed using the 3,3′,5,5′-Tetramethylbenzidine (TMB) substrate reagent (Becton Dickinson Biosciences, Franklin Lakes, NJ). Plates were read on a Bio-Rad model 2550 plate reader (Bio-Rad, Hercules, CA).

### 2.9 Isolation and stimulation of lymphocytes

Mice were vaccinated followed by a boost two weeks later. One week following boost, mice were either unchallenged or challenged with *Lm + MAX*. One week later (4 weeks after initial vaccination) the popliteal and/or paraaortic/lumbar lymph nodes (LN) were harvested (*n* = 5–8) and mechanically disrupted to prepare separate single-cell suspensions in complete RPMI medium. 1 × 10^6^ cells were added to each well of a 48-well plate in a volume of 500 µL of complete RPMI medium. 1 µL of both Cell Stimulation Cocktail (500x, eBioscience, San Diego, CA) and GolgiPlug™ Protein Transport Inhibitor (BD Biosciences Pharmingen, San Diego, CA) were added to each well, and cells were then incubated for 5 h at 37 °C. Cells were processed according to manufacturer’s protocol.

### 2.10 Intracellular cytokine staining and flow cytometry

Following stimulation, cells were suspended in FACS staining buffer (PBS, 0.5% BSA, and 0.01% azide) and treated for 15 min with Fc receptor block (Miltenyi Biotec, San Diego, CA) and surface-labeled with PE- or APC-conjugated Abs for 20 min at 4 °C. Cells were permeabilized, stained and fixed using Cytofix/Cytoperm™(BD Biosciences San Diego, CA) according to manufacturer’s protocols. Intracellular cytokines (IFNγ or IL-4) were stained using FITC-conjugated antibodies and analyzed for expression of cytokines by flow cytometry, (CyAn flow cytometer, DakoCytomation, Fort Collins, Colorado) using Summit Acquisition Software, Version 4.2.

### 2.11 Isolation of DCs from in vitro cultures of bone marrow cells

Cultures of bone marrow cells from BALB/c or C3H mice were established as described [4], [19]. Following 5–7 days in GM−CSF/IL-4 culture, cells were harvested and CD11c^+^ cells were purified using the magnetic bead isolation kit from Miltenyi Biotec (San Diego, CA). Bone marrow-derived- (BM)-DCs were treated with either vehicle (PBS), 10 ng/ml MAX, 10 ng/ml MAX pretreated with 1% anti-MAX antisera, 7 ng/ml PACAP_6-38_ (PAC1 antagonist) or 5 ng/ml OVA_258-265_ SIINFEKL for 3 h. DCs were subsequently treated with 500 ng/ml lipopolysaccharide (LPS) for 36 h and expression of CD80 and CD86 were determined by flow cytometric analysis as described above.

### 2.12 Statistical analysis

Statistical analyses were conducted using Prism 7.0 software (GraphPad, La Jolla, CA). Data for lesion progression were analyzed using ANOVA for repeated measure with Tukey’s multiple comparisons test. For comparisons between two groups, two-tailed *t* tests were performed. Data were considered statistically significant for *p* < .05.

## 3 Results

### 3.1 Footpad lesions show comparative improvement in Lm+MAX-challenged mice vaccinated with CLDC containing peptides from FL MAX or 15 AA peptides from N- or C- terminal regions of MAX

**Fig. 1:**
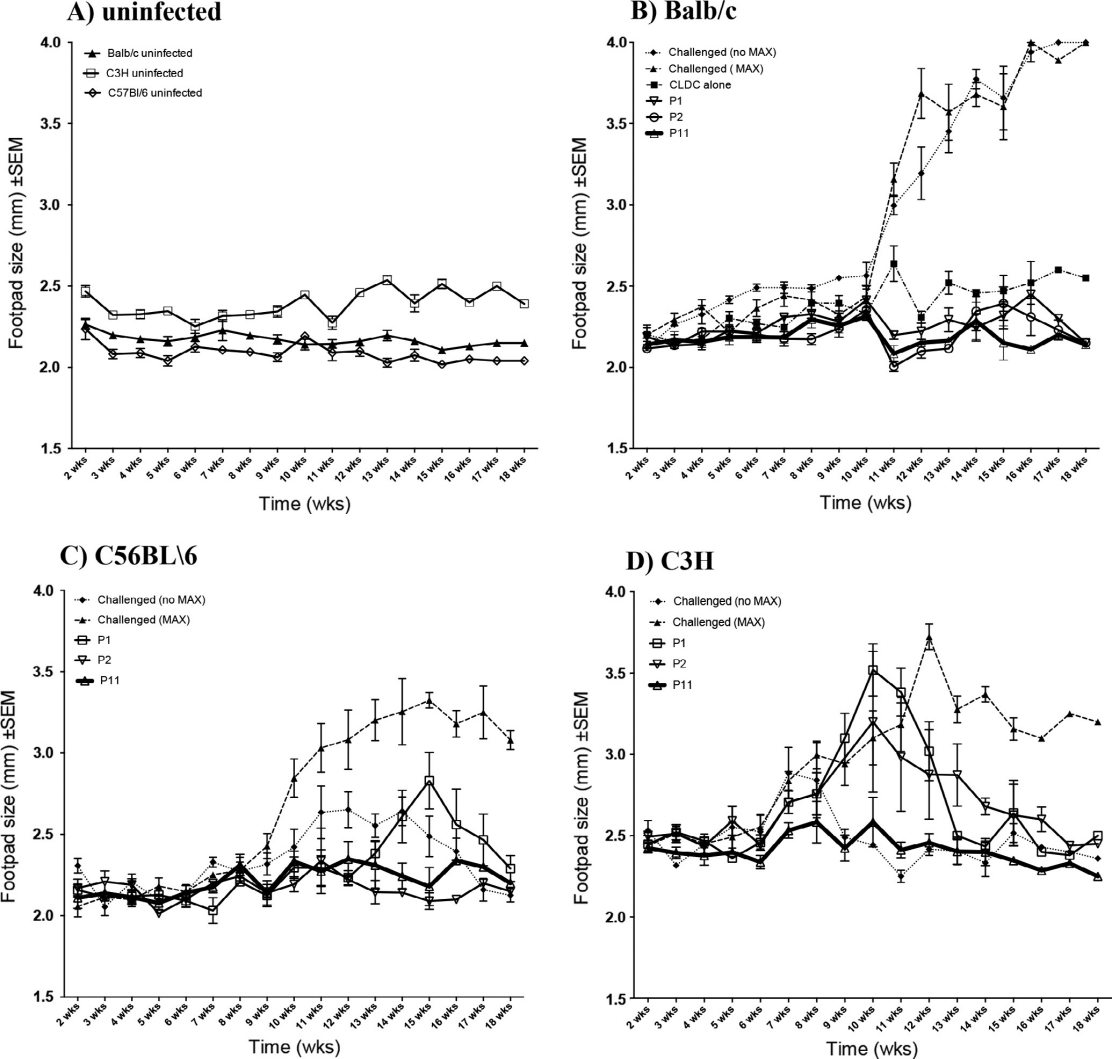
Vaccination with CLDC containing 15 AA peptides, encompassing the N- and C-terminal domains of MAX (P1, P2 and P11) protects three strains of mice from footpad swelling when challenged with *Lm* + MAX compared to unvaccinated animals. (A) All three strains of mice were sham vaccinated with vehicle (5% tris-buffered dextrose) and unchallenged and footpad measurements were taken weekly for 18 weeks to determine any increase in pad size due to the overall growth of the animals over the period. (B–D) Changes in footpad size over and 18 week period was determined for (B) Balb/c, (C) C57BL/6 and (D) C3H mice that were either challenged with 100–1000 *Lm* metacyclic promastigotes alone (“Challenged (no MAX)”; dotted lines, solid diamonds), or challenged with *Lm* + MAX (“Challenged (MAX)”; dashed lines, solid triangles), or challenged with *Lm* + MAX after being vaccinated/boosted with CLDC prepared with either the P1 peptide (solid line, open inverted triangles), P2 peptide (solid line, open circles) or P11 peptide (thick solid line, open triangles). All mice were challenged two weeks after the boost. Additionally, mice were sham vaccinated with the CLDC adjuvant alone (without MAX) and challenged with *Lm* + MAX (dot-dash line, solid squares in panel B) Sham vaccination/boost with CLDC adjuvant alone was also performed on C57BL/6 and C3H mice but is not shown since there was no significant differences in footpad swelling when compared to the unvaccinated *Lm* + MAX challenged animals. Lesions were measured over an 18 week period using Vernier^®^ calipers. Two independent investigators who were blinded from the treatment types performed the measurements.

**Fig. 2:**
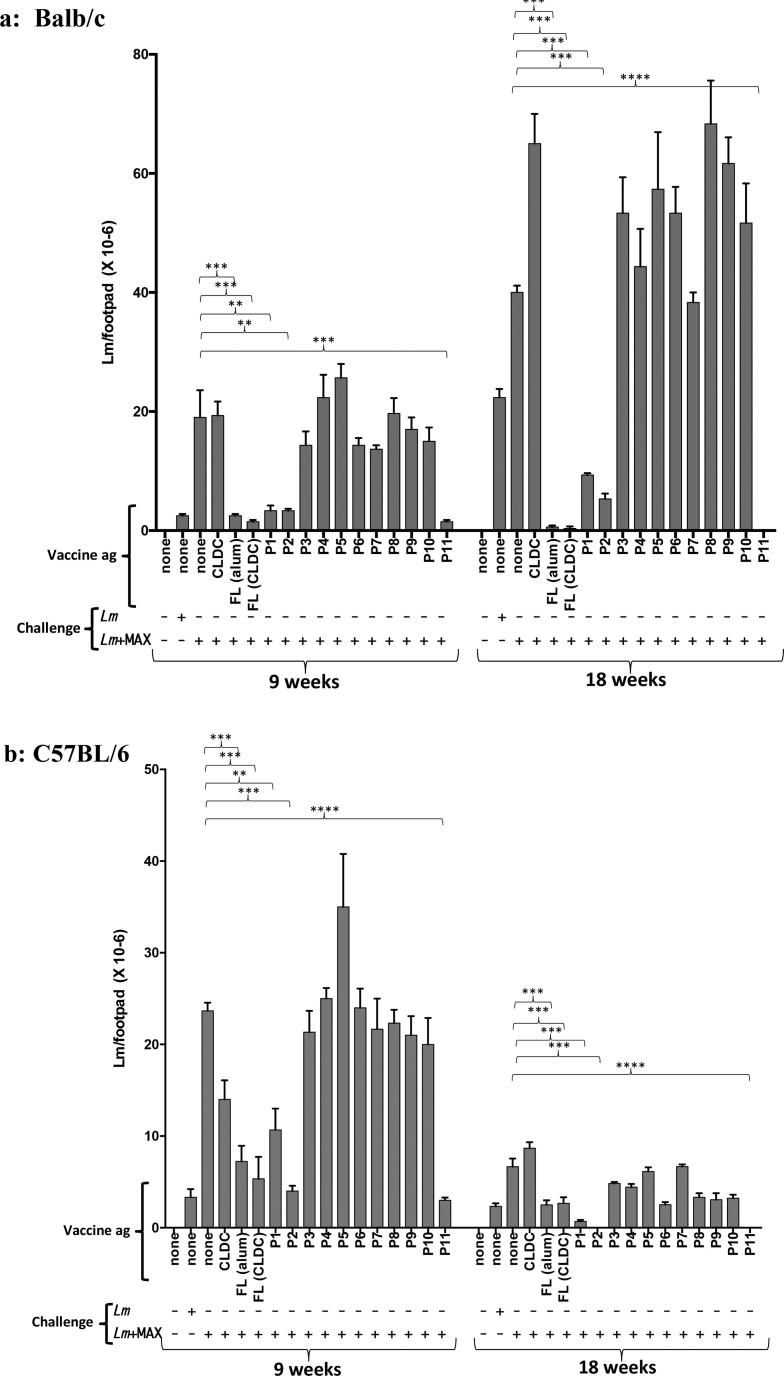

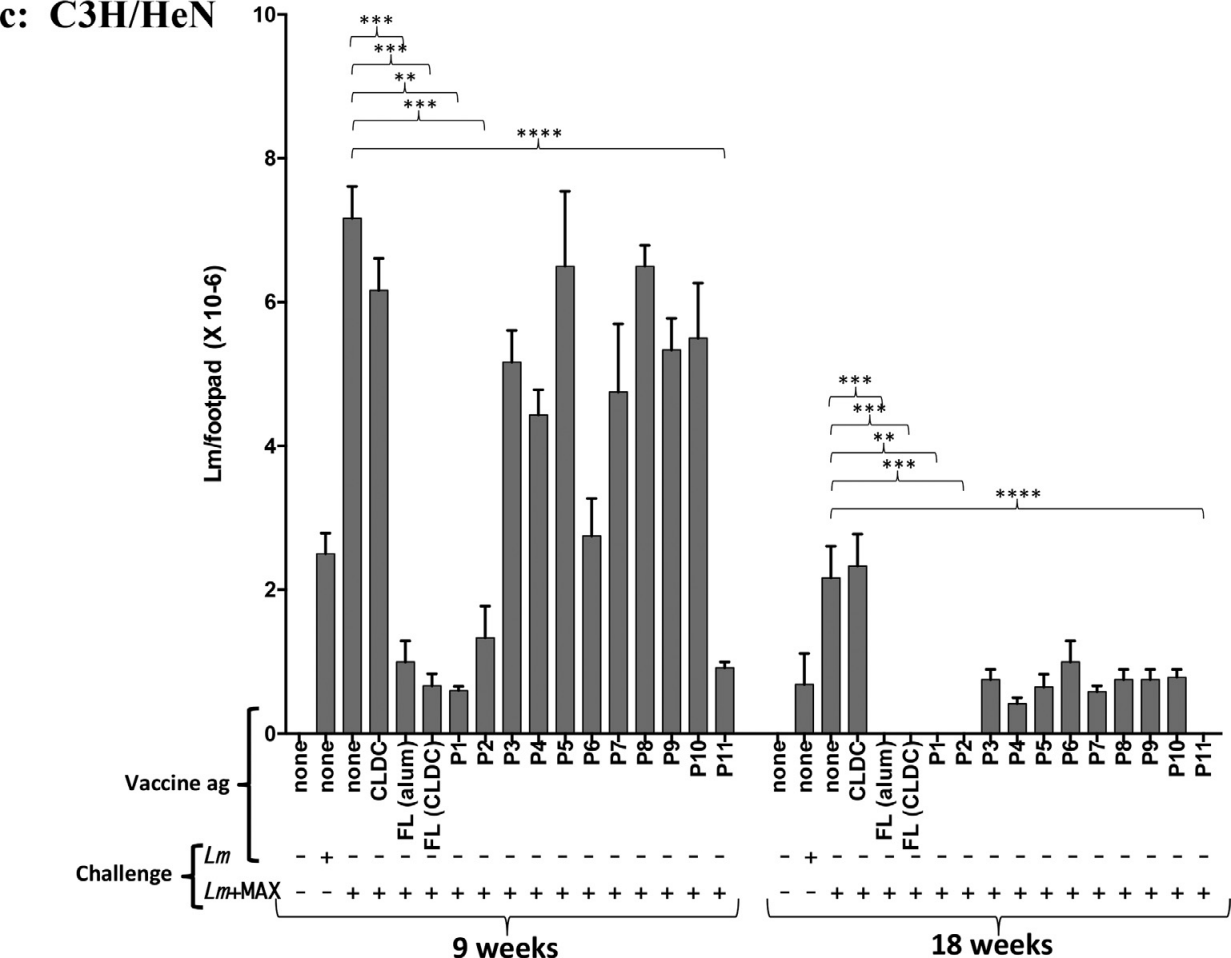
Vaccination of (2a) BALB/c, (2b) C57BL/6 or (2c) C3H-HeN mice with CLDCs containing FL-MAX, P1, P2 or P11 resulted in a significantly reduced parasite burden in extracted footpads 18 weeks post low-dose (10^2^-10^3^) *Lm*+MAX challenge. Mice were vaccinated/boosted with CLDC admixed with either FL MAX or 11 overlapping peptides encompassing the 63 amino acid MAX peptide. Control mice were either; i) unchallenged, ii) challenged with *Lm* alone and unvaccinated, iii) challenged with *Lm*+MAX and unvaccinated or iv) challenged with *Lm* +MAX and vaccinated with CLDC without antigen. FL-MAX-Alum and FL-MAX-CLDC vaccine formulations are designated “FL(Alum)” and “FL(CLDC)” in the figure. Two mice of each treatment group were euthanized at 9 weeks post-challenge and 3 animals likewise at 18 weeks post-challenge. Footpads were removed and homogenized. Parasite numbers were determined by limited dilution analysis. The bars represent the average of 2 and 3 mice per treatment group for 9 and 18 weeks post *Lm* challenge respectively. Results are representative of three repeat experiments. Error bars represent the mean ± SEM for the treatment/vaccination groups. P-values were calculated for statistical variance using a paired two-tailed Mann-Whitney test. (“ns”: not significant; *, p < .05; **, p < .01; ***, p < .005 and ****, p < .001). P values are shown for comparisons between *Lm*+MAX and P1, P2 and P11 vaccinated *Lm*+MAX challenged mice.

**Supplementary Fig. 3:**
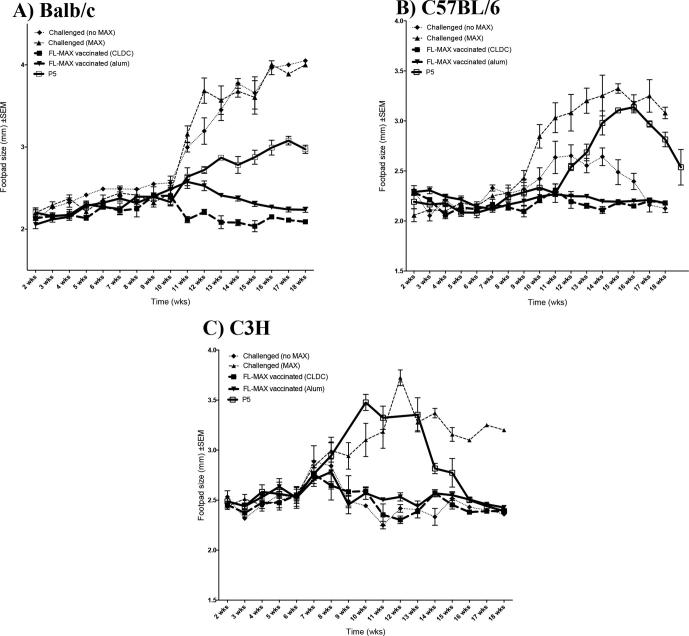
Vaccination of mice with P5-CLDC failed to improve lesion induration over the 18 week course after MAX + *Lm* challenge and immunization of BALB/c mice with FL-MAX (CLDC) yielded significantly better lesion healing verses the FL-MAX(alum) formulation as assessed by lesion size. Footpad size was determined for (A) Balb/c, (B) C57BL/6 and (C) C3H mice that were either challenged with 100-1000 *Lm* metacyclic promastigotes alone (dotted lines, solid diamonds), challenged similarly with *Lm* admixed with 10 ng MAX (dashed lines, solid triangles), or challenged with *Lm* + MAX after being vaccinated/boosted with CLDC prepared with the non-efficacious P5 peptide(solid thick line, open squares), FL-MAX-alum (solid thick lines, inverted solid triangles) and FL-MAX-CLDC (thick solid dashed line, solid squares). All mice were challenged one week after the boost. Lesions were measured over an 18 week period using Vernier^®^ calipers. Two independent investigators who were blinded from the treatment types performed the measurements.

MAX-CLDC vaccine candidate antigens were comprised of either synthetic full-length (FL) MAX or short 15 AA peptides spanning the 63 AA length of MAX. Synthetic peptides are illustrated in Supplemental Fig. S1. BALB/c, C57BL/6 and C3H-HEN mice were vaccinated and boosted separately with 12 MAX-CLDC candidate vaccines (the Full-length (FL) MAX and 11 MAX peptide vaccine formulations) (n = 5 mice per vaccine candidate)). Fig. 1 shows the comparative kinetics of lesion swelling over 18 weeks between P1, P2 and P11 vaccinated and unvaccinated control mice (compare solid lines, open circles and open triangles in Fig. 1, panels B, C and D for P1-, P2- and P11-CLDC results). P1-, P2- and P11-CLDC vaccines were efficacious in terms of reduced lesion size. The P11-CLDC vaccine consistently prevented the formation of severe footpad lesions throughout the course. Sham vaccination with CLDC alone in some cases prevented maximal swelling of footpad size; however there was no corresponding decrease in parasite burden in these mice (compare Fig. 1 panel B, (dot-dash line solid squares) with Fig. 2). Mice vaccinated with FL-MAX/CLDC were protected from lesion swelling (Supplementary Figs. S2 and S3). However, those vaccinated with FL-MAX alone were not protected (Data not shown.) MAX + *Lm* challenged-mice using CLDC vaccines containing P3-P10 formulations did not demonstrate significant differences in footpad swelling when compared to those that were unvaccinated for all 3 strains of mice (Fig. S3 (for peptide P5) and data not shown).

### 3.2 Reduced parasite burden in footpads from peptide-vaccinated mice 9 and 18 weeks post low-dose Lm+MAX challenge

**Supplementary Fig. 4:**
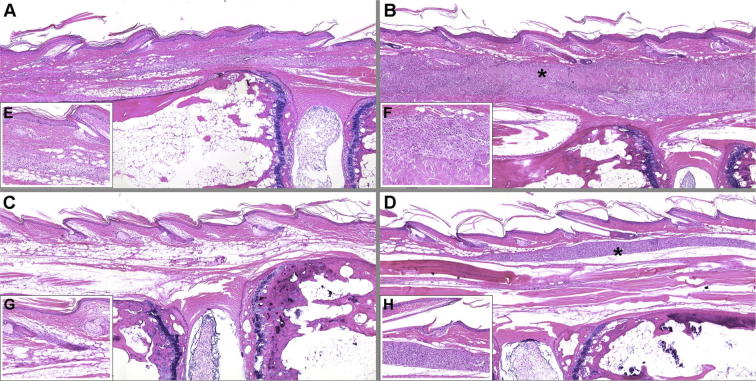
The base-of-tail immunization site of mice treated with Alhydrogel^®^ adjuvant contains a severe inflammatory infiltrate that is absent at the immunization site of mice treated with CLDC adjuvant. BALB/c mice (*n =* 5 animals) were immunized s.c. at the base of the tail with MAX using either Alhydrogel^®^ or CLDC as adjuvant, and were challenged subcutaneously in one hind footpad with 1 x 10^3^*Lm* metacyclic promastigotes admixed with 10 ng synthetic FL-MAX. At various time points following immunization, tails were harvested, sectioned, and H&E stained for evaluation of histopathologic changes at the site of immunization. Direct comparison of representative tail sections of CLDC-adjuvanted (A & E) vs. Alhydrogel^®^-adjuvanted mice (B & F) 14 days post-boost reveals the presence of a severe inflammatory infiltrate (asterisk) at the site of immunization in the Alhydrogel^®^-adjuvanted group, which is minimal to absent in the CLDC-adjuvanted group. By 245 days post-boost, the tail section from the CLDC-adjuvanted group (C & G) appears normal in comparison to tail sections from un-immunized control mice (not shown), while the severe inflammation persists within the Alhydrogel^®^-adjuvanted group (D & H). Images are representative of results obtained from three independent experiments. Overview: 10x magnification, inset: 20x magnification.

At 9 weeks and 18 weeks post *Lm* + MAX challenge, mice were euthanized and footpads were removed and the number of parasites/footpad was determined. In all challenged/unvaccinated mice parasite burden was initially high. Fig. 2a and 2b show that, in the *Lm* susceptible BALB/c strain and the healing C57BL/6 strain, parasite burden is apparent after 9 weeks post-challenge with either *Lm* or *Lm* + MAX while overall parasite burden is comparatively low in C3H mice (Fig. 2c). Co-injection of *Lm* + MAX resulted in increased parasite burden in mice compared to those challenged with *Lm* alone. At 9 weeks post-challenge, when compared to challenge with *Lm* alone, MAX + *Lm* challenges resulted in averages of 10-fold, 5-fold, and 3-fold increases in parasite burden in BALB/c, C57 and C3H mice, respectively. Moreover, all strains of mice immunized with CLDC containing P1, P2, or P11 Ag peptides demonstrated decreases in parasite burden after 9 or 18 weeks post challenge when compared to unvaccinated controls (Fig. 2). For all three strains, the most protective CLDC adjuvanted vaccines were those containing either the FL MAX or the P11 peptide. Vaccination with CLDC containing an irrelevant protein antigen (Hen egg lysozyme (HEL) did not improve parasite burden in any mouse strain (Data not shown). Although FL-MAX-Alum proved to be efficacious, there was considerable inflammation at the injection site that persisted for over 240 days post-injection. This was not the case for the CLDC formulation (Supplemental Fig. S4).

### 3.3 Serum antibodies from BALB/c, C57BL/6 and C3H immunized with the P11 CLDC recognize both FL MAX and the P11 peptide and neutralized MAX-mediated DC reprograming in vitro

**Fig. 3:**
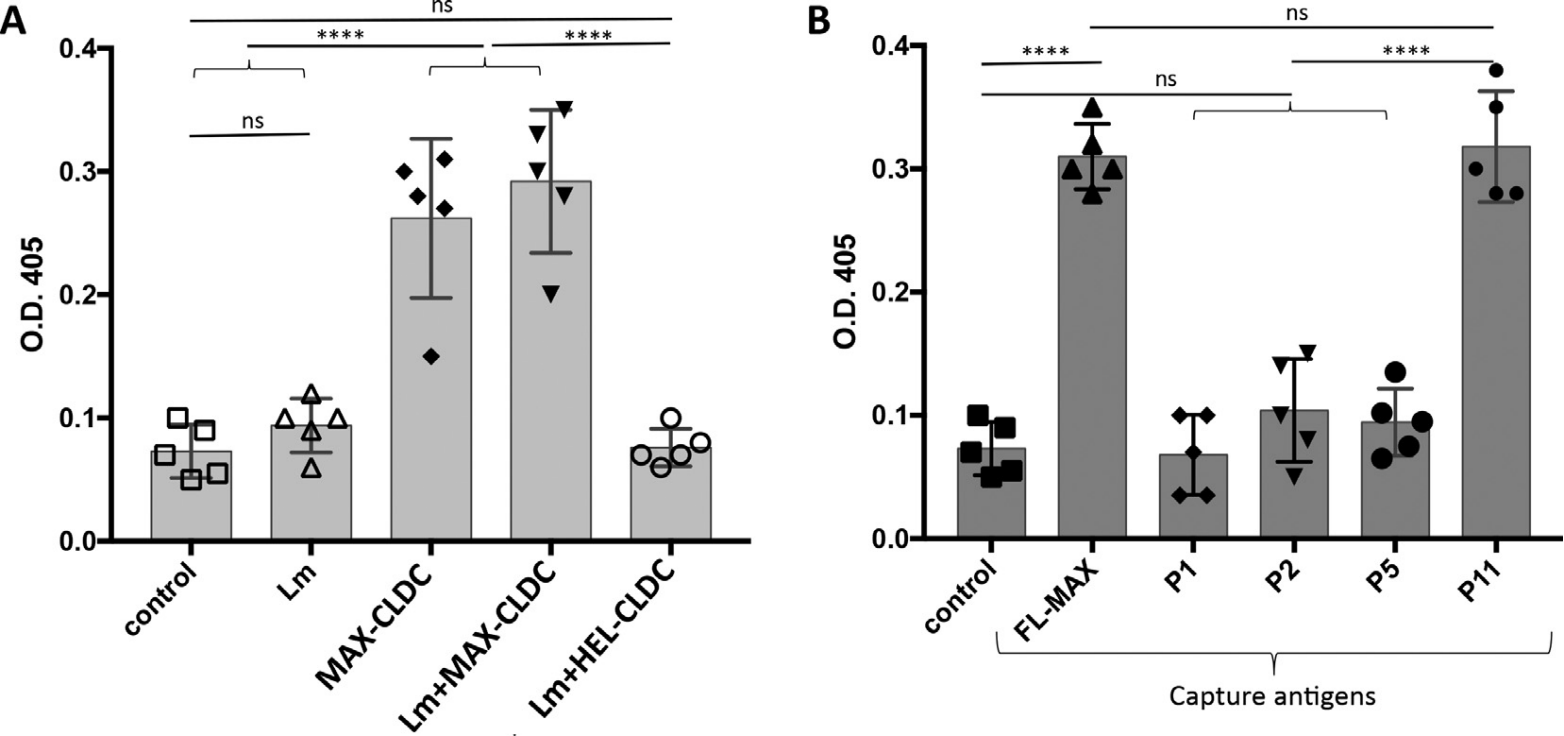
A. Antisera (diluted 1:100) from BALB/c mice vaccinated with FL-MAX CLDC (either challenged with *Lm* + MAX or not) recognizes synthetic MAX as capture antigen. Antisera were evaluated by ELISA from mice that were either unvaccinated and not *Lm* challenged (control), unvaccinated but *Lm* challenged (Lm) or vaccinated with CLDC-MAX and not *Lm*+MAX challenged (MAX-CLDC), MAX-CLDC vaccinated and *Lm*+MAX challenged (Lm+MAX-CLDC) or vaccinated with HEL-CLDC and *Lm*+MAX challenged (*Lm*+HEL-CLDC). B. Sera from BALB/c, mice immunized against the P11 C-terminal peptide of MAX using P11-CLDC have antibodies that exclusively recognize the P11 peptide or the FL MAX molecule. Animals were immunized against the MAX P11 peptide CLDC as described in the Materials and Methods section and were analyzed by capture ELISA using, BSA (control), FL-MAX or the P1, P2, P5 or P11 MAX peptides (referenced in supplemental Fig. S1) as capture antigens. Error bars represent the mean O.D._405_ between five individual animals. P-values were calculated for statistical variance using a paired two-tailed Mann-Whitney test. (“ns”: not significant; *, p < .05; **, p < .01; ***, p < .005 and ****, p < .001).

**Fig. 4:**
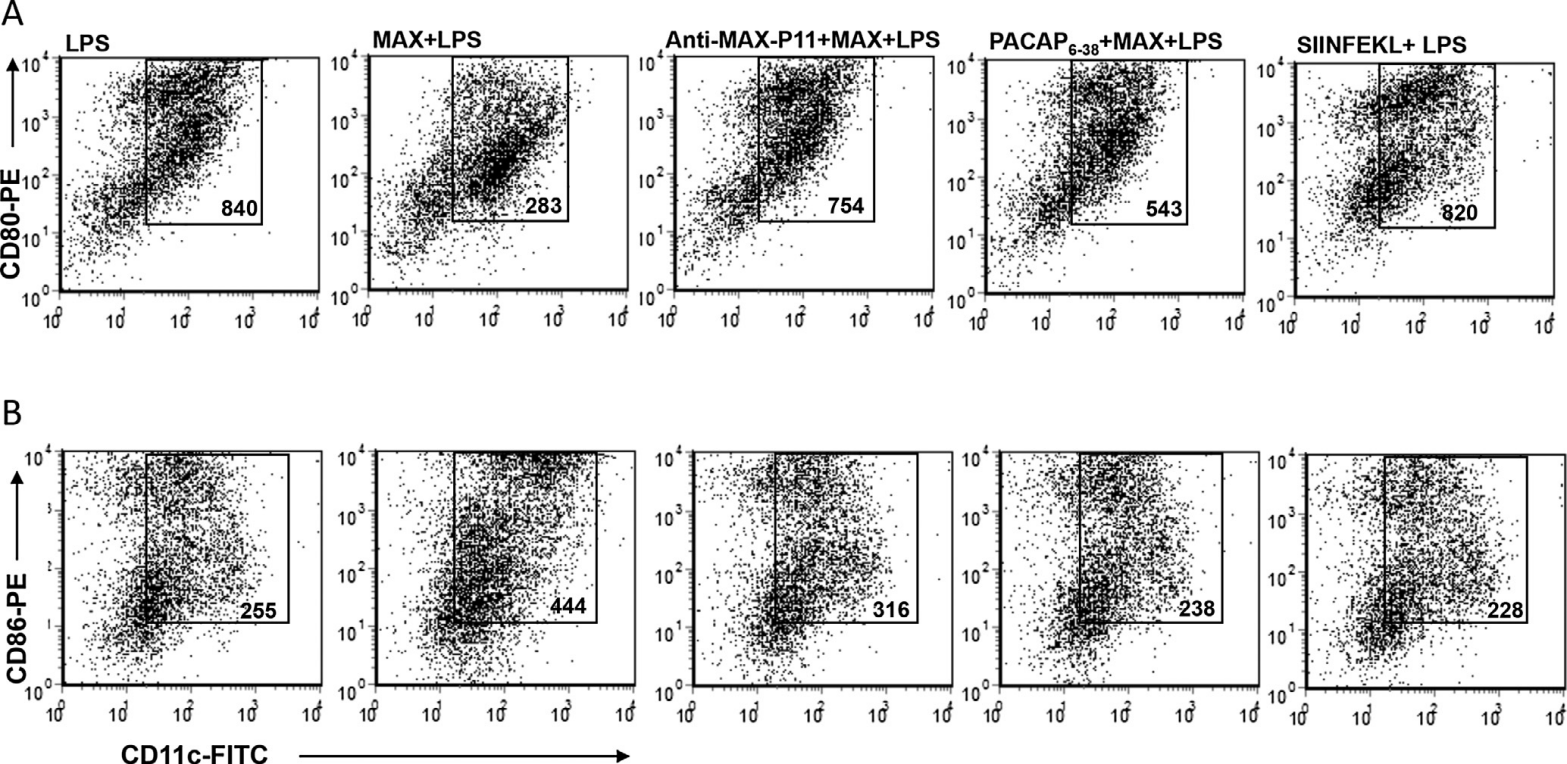
Antisera from P11-CLDC-vaccinated C57BL/6 mice challenged with *Lm* + MAX blocks the MAX-mediated reprogramming of LPS-activated BM-DC. BM-DCs from C57BL/6 mice were treated for 3 h with either 10 ng of synthetic MAX or 10 ng of MAX pretreated with a 1:100 dilution of serum from P11-CLDC vaccinated mice. The DCs were subsequently treated with 500 ng/ml LPS for 36 h. As controls, DCs were pretreated with either the PAC1 receptor antagonist PACAP_6-38_ (12 ng) or an irrelevant peptide (OVA_258-265_-SIINFEKL) followed by stimulation with 500 ng/ml LPS for 36 h. DCs were harvested and stained with CD11c-FITC, and either CD80-PE (A) or CD86-PE (B) and analyzed by flow cytometry. The numbers inside the boxes are the geometric Mean Fluorescent Intensity (g-M.F.I.) of gated CD11c^+^ events. Results are representative of 3 experiments for each mouse strain.

Sera from mice vaccinated with CLDC and FL MAX- or C— or N-terminal MAX peptides contained detectable titers of anti-MAX immunoglobulin at 18 weeks following immunization (Fig. 3A for Balb/c and data not shown). Sera from FL-MAX-CLDC immunized mice contained antibodies that recognized primarily FL-MAX, P1, P2 and P11 MAX peptides as capture antigens (Data not shown). Serum ELISA from P11-CLDC-immunized Balb/c mice indicated that antibodies are elicited in response to this vaccine and are restricted to recognition of the FL-MAX and P11 epitopes (Fig. 3; panel B). This was the case for all P11-CLDC vaccinated mice (not shown). These results in combination with lesion analysis and the parasite burden data suggest that the antibodies recognizing the P1, P2 and P11 regions of MAX likely contribute to a protective effect. In contrast, sera from mice vaccinated with CLDC deploying P3-4 and P6-10 failed to detect plate-bound MAX in ELISA assays whereas the P5 formulation yielded an antibody titer that had no efficacy (Data not shown, Fig. 2 and supplementary Fig. S3). Additionally, treatment of synthetic MAX with anti-MAX P11 (1%) antisera prior to addition to in vitro cultures of BM-DCs effectively blocked the MAX effect of abrogating the up-regulation of CD80 on LPS-stimulated BM-DCs as previously observed [4] (Fig. 4). In addition, sera from MAX-P11-CLDC vaccinated mice blocked the MAX-mediated up-regulation of CD86 on LPS-stimulated BM-DCs. As controls, BM-DCs were pretreated with the type 1 pituitary adenylate cyclase-activating peptide (PAC1) receptor antagonist, PACAP_6-38_, prior to MAX treatment. PACAP_6-38_ partially blocked the MAX effect on CD80 expression on BM-DCs from C57BL/6 mice (Fig. 4A, CD80 panel). However, PACAP_6-38_ effectively blocked the MAX mediated up-regulation of CD86 (Fig. 4B, CD86 panel). MAX has been shown to interact and signal through PAC1 and likely mediates its immunosuppressive and anti-inflammatory effects thereof. As a control for the PACAP_6-38_ treatment, DC pretreated with ovalabumin_258-265_ peptide, SIINFEKL, did not affect the LPS + MAX response on BM-DCs (Fig. 4).

### 3.4 MAX-CLDC immunized mice challenged with Lm + MAX have increased percentages of IFNγ-secreting and decreased IL-4-secreting CD4^+^ cells from footpad draining lymph nodes (LNs)

**Fig. 5:**
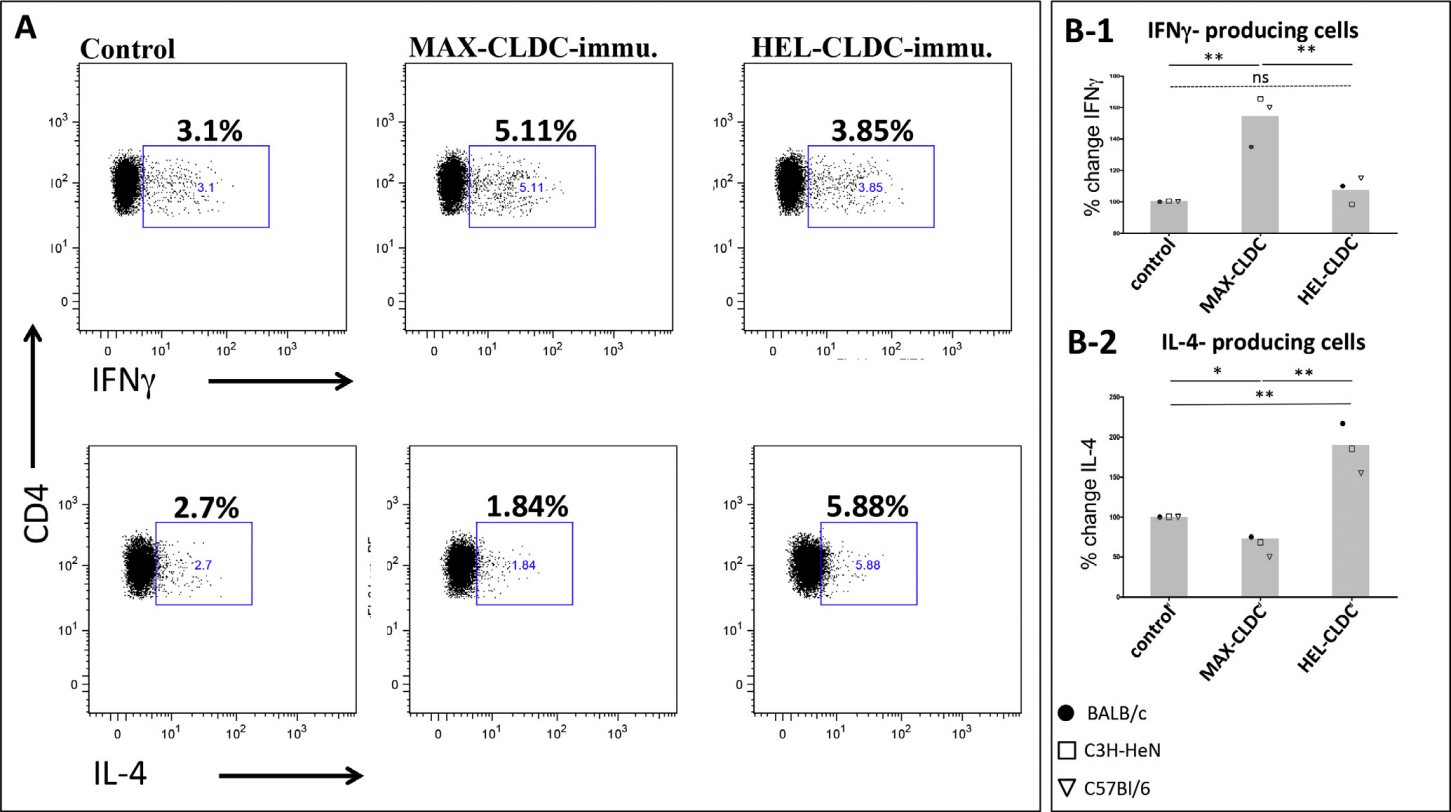
Intracellular staining of IFNγ- and IL-4-producing CD3/CD4^++^ cells from *Lm* challenged unvaccinated, MAX-CLDC or HEL-CLDC immunized mice. Footpad-draining popliteal LN were isolated from mice (n = 3–5 animals) vaccinated and boosted with either FL-MAX-CLDC, or HEL-CLDC and challenged one week later with low-dose MAX+ *Lm*. CD4+ cells were evaluated for intracellular IFNγ and IL-4. Single cell suspensions were stimulated in vitro, surface stained for CD3 and CD4 followed by intracellular staining for IFNγ or IL-4. (A) Flow cytometric analysis of CD3/CD4^++^ events revealed an increase percentage of IFNγ-producing cells in LN from MAX-CLDC-immunized BALB/c mice. Similar analysis of IL-4-producing cells demonstrated a decrease in IL-4-producing CD3/CD4^++^ cells in response to *Lm*+MAX challenge. (B) Summary plots from cumulative analysis of all three strains of mice (each point is a plotted average percentage of 3 animals per strain). Values are expressed as percent change from an assigned percentage (100%) of control mice. Results are representative of 3 separate experiments (i.e., a total of 6 mice analyzed per strain for each treatment group). P values for statistical variance were determined using repeated measures ANOVA with Tukey’s multiple comparison tests. (* p < .05, ** and p < .01).

One week post-challenge with *Lm* + MAX, footpad-draining (popliteal (or paraaortic/lumbar)) LNs were harvested from mice (n = 3–5 animals) that were previously unvaccinated (control), or immunized with either FL-MAX-CLDC or CLDC admixed with hen egg lysozyme (HEL-CLDC). Single cell suspensions were stimulated in vitro as described above, surface stained for CD3 and CD4 followed by intracellular staining for IFNγ or IL-4. Flow cytometric analysis of CD3/CD4^++^ events revealed an increased percentage of IFNγ-producing cells in LN from MAX-CLDC immunized animals from 3.1% to 5.11% representing an approximate 65% overall increase in IFNγ-producing cells (Fig. 5; Panel A). Similar analysis of IL-4-producing cells demonstrated a decrease from 2.7% to 1.84% representing an approximate 45% decrease in IL-4-producing CD4^+^ cells in response to *Lm* + MAX challenge (Fig. 5; panel A). Analyses of all three strains of mice were plotted as a function of the average percent change of 3 mice from each strain from standard control results (arbitrarily set at 100%) from unvaccinated mice (Fig. 5; panel B-1 and B-2). The overall increased percentage ratio of IFNγ- vs. IL-4-producing CD4^+^ in MAX-CLDC-immunized mice was 2.53. Whereas ratio of IFNγ- vs. IL-4-producting CD4^+^ cells was only 1.13 in non-immunized animals, suggesting an increased Th1-bias in all strains due to immunization with CLDC+MAX. The Th1-biased immune response is potentially capable of protecting against intracellular *Lm* infection; thus, the pattern of cytokine production identified in the draining lymph nodes of MAX-immunized mice challenged with *Lm* + MAX might, in part, account, for the protection induced against challenge.

## 4 Discussion

We demonstrated that FL-MAX, C- and N- terminal peptides thereof can be utilized as Ag, without parasite components, in a liposome/DNA adjuvant vaccine system protecting three strains of mice from potentiation and exacerbation of *Lm* infection. The C- and N-terminal portions of MAX, have been shown to be important for either receptor binding or functionality [20], [21]. MAX may skew dermal DCs and macrophages towards Type 2 immune responses rendering hosts more vulnerable to *Lm* infection [4], [22], [23], [24]. The low-dose challenges increased the time for the development of relatively minor lesions in C3H and C57BL/6 mice. However this approach was proper for these experiments in order to best recapitulate natural infection processes. Parasite inoculums were at sand fly-carrying levels reported to be about 10–1000 parasites per inoculum [25], [26], [27]. These results also indicate the feasibility of generating a more affordable and easily manufactured anti-MAX vaccine by virtue of deploying small peptides.

Significantly, very few or no parasites were detected in MAX-CLDC or P1, P2 and P11-CLDC vaccinated BALB/c mice footpads 18 weeks post-challenge. This was consistent for two separate experiments spanning a total of 10 mice. This poses a conundrum since BALB/c mice are susceptible to *Lm* regardless of whether vectored by sand flies or co-injected with either salivary gland extract (SGE) or MAX. It is possible that the adjuvanted anti-MAX response primes host immunity to provoke Th1 responses necessary to resolve low-dose infection.

It is important to note that *Lm* is naturally transmitted by Old World sand flies such as *P. papatasi* and *P. duboscqi* not by the New World fly, *Lu. longipalpis*. Hence this study does not use a salivary component from natural vectors for *Lm* and thus not strictly mimicking the natural infective process by pairing the appropriate sand fly/parasite combination. Rather, we demonstrate the feasibility of generating a protective host immune response against MAX, a potent disease exacerbative component of *Lu. longipalpis*. Salivary components increase parasite infectivity using various vector/pathogen combinations in vivo: *Lu. longipalpis* and *Lm* [27], *P. papatasi* and *Lm* [28], [29], [30], *Lu. longipalpis* and *Leishmania donovani chagasi* [31], and *Lu. longipalpis* and *L. amazonensis* [32]. Thus, exposure to vector salivary components alter host hemostasis and/or immune responses, suggesting these mechanisms are general and conserved in nature. Although MAX is absent from *P. papatasi* saliva, activities such as vasodilation and immune modulation that are related to MAX have been attributed to a variety of Old Word sand fly salivary molecules [2], [33], [34]. MAX was deployed as Ag in this study because it is better biochemically characterized, and thus more suited to analysis.

In the current study, the overall percentage ratio of IFNγ- vs. IL-4-producing CD4^+^ cells in the draining LN of MAX-CLDC-immunized mice was 2.53, in comparison to a ratio of 1.13 in non-immunized mice, suggesting an increased Th1-bias. The increased amount of IFNγ-secreting cells likely contributes to the protection. It has been postulated that antibodies play no role in saliva-mediated protection [35], [36]. We hypothesize otherwise that anti-MAX Abs may serve to neutralize the potentiation effect of MAX thereby establishing transmission of the parasite in a more protective Th1 microenvironment.

Since no *Lm* Ags were used in the vaccine formulations, the platform described herein is not entirely prophylactic and *Lm* infection is initially established in all three strains of mice. Rather, the established immunity to this salivary component may prevent the reprograming of innate immune responses permitting a more protective host cellular response against parasite transmission, growth and persistence [4], [5]. Combinatorial vaccines that incorporate antigens of various *Leishmania spp*. as well as their cognate salivary potentiators would likely provide the best components for an eventual efficacious prophylactic vaccine [37].

When combined with nucleic acid agonists, the CLDC adjuvant offers immune potentiation and TLR ligands for endosomally located TLRs (TLR3, TLR7/8 and TLR9) and cytosolic nucleic acid receptors (e.g. RIG-I and DAI [16], [38], [39], [40], [41], [42], [43]. Indeed, in the current study, we demonstrate that CLDC-adjuvanted MAX performed better than alum in terms of lesion induration, parasite burden and inflammation at the injection site (Fig. 2, Supplemental Figs. S3 and S4).

In nature, *Leishmania* species are antigenically diverse confounding efforts to producing a single vaccine for disease control. Additionally, development of a universal anti-MAX vaccine has been hampered by concerns that considerable variation of MAX exists in nature. Variation has been speculated as being one of the adaptive mechanisms that sand flies have evolved to survive host immune responses generated by repeated biting [44]. While it is clear that variations of MAX exist, there are peptide domains that must remain conserved in order to fulfill requisite functions. Among known natural MAX variants, the 15 AA (P11) C-terminal domain is somewhat conserved with only a few AA substitutions that are themselves conservative. Currently, there are only 4 known natural variants [13]. This conservation has been demonstrated by the construction of MAX deletion mutants showing that the C-terminal domain is absolutely required for receptor binding [21]. Hence, antibodies targeting this domain would likely act as antagonists to MAX receptor binding. The PAC1 receptor for MAX is expressed on a variety of cell types including, neurons, endothelial cells, macrophages, and DC [45]. The human ligand for PAC1, PACAP, is a neuropeptide and is involved in neurotransmission, vasodilation and various endocrine effects [45], [46]. PACAP has no structural or sequence similarity to MAX so antibodies generated against MAX likely won’t cross-react with PACAP and negatively affect its binding to PAC1 [21]. Indeed antibodies to MAX exist in the serum of dogs and people endemic to areas populated by *Lu. Longipalpis*. As a rule, sand flies are not strong fliers and, as such, local populations of *Lu. Longipalpis* which have large geographical distributions throughout the New World are likely to be genetically isolated [47]. Thus development of “regional” vaccines may be necessary. Hence, in developing such vaccines, perhaps entertaining the notion of tailored vaccines enhanced by salivary components of regionally-specific vectors is warranted.

## Conflict of interest statement

None declared.
